# An investigation of higher order moments of empirical financial data and their implications to risk

**DOI:** 10.1016/j.heliyon.2022.e08833

**Published:** 2022-02-03

**Authors:** Luke De Clerk, Sergey Savel'ev

**Affiliations:** Department of Physics, Loughborough University, Leicestershire, LE11 3TU, United Kingdom

**Keywords:** Empirical data, Scaling relations, Higher order standardised moments, Value-at-risk, Gaussian mixtures

## Abstract

Here, we analyse the behaviour of the higher order standardised moments of financial time series when we truncate a large data set into smaller and smaller subsets, referred to below as time windows. We look at the effect of the economic environment on the behaviour of higher order moments in these time windows. We observe two different scaling relations of higher order moments when the data sub sets' length decreases; one for longer time windows and another for the shorter time windows. These scaling relations drastically change when the time window encompasses a financial crisis. We also observe a qualitative change of higher order standardised moments compared to the gaussian values in response to a shrinking time window. Moreover, we model the observed scaling laws by analysing the hierarchy of rare events on higher order moments. We extend the analysis of the scaling relations to incorporate the effects these scaling relations have upon risk. We decompose the return series within these time windows and carry out a Value-at-Risk calculation. In doing so, we observe the manifestation of the scaling relations through the change in the Value-at-Risk level.

## 1 Introduction

In many financial settings, the behaviour of market data is analysed to better understand: the logarithmic price change, [1], [2], [3], [4], the historic or implied volatility, [5], [6] or the actual price behaviour [7], [8], [9]. Nevertheless, in [10], the higher order moments were used to study the applicability of certain Generalised AutoRegressive Conditional Heteroskedasticity (GARCH) models for mimicking price dynamics. The use of higher order moments within financial modelling is well established, [11]. By investigating the higher order moments we can get an insight to the distribution of price change and how it varies over time. By doing this, we can evaluate the hypothesis that rare-events originate from huge volatility shocks, as such this phenomenon is likely seldom seen in short time windows and is much more likely in long time windows. This observation helps us to understand the behaviour of higher order central moments in different time windows.

The higher order moments are used in this investigation due to their ability to capture the general aspects of the distribution of price change, [12], [13], [14]. The higher order moments show the quantity of outliers within the distribution, [11]. If the fourth order standardised statistical moment (also called kurtosis) of empirical data sets is larger than 3, we have a leptokurtic distribution. This manifests itself in a higher probability of getting an outcome that is much larger or smaller than the mean. Such a behaviour is also known as a rare-event. Therefore, we can study the properties of the time series without the need for many different metrics.

Higher order moments are used in various applications for analysis of financial assets and derivatives. As is well known, if all moments of a stochastic process are known, the probability distribution of a random variable can be reconstructed. Therefore, the more moments known, the better the estimation of the distribution we can obtain. In the case of option and derivative pricing, [15], [16], [17], the Gram-Charlier Type A expansion, which uses higher order moments, is used to recover the probability distribution of the process to enable accurate pricing of derivative instruments. Furthermore, in deducing an investor's utility function within the Markowitz mean-variance portfolio theory, a Taylor expansion of the utility function is needed to gain accurate inferences of the risk an investor is willing to accept, [18]. To increase accuracy of such a task, the evaluation of higher order statistical moments is critically important. Additionally, higher order moments (third and fourth order standardised moments) have been used within the decomposition of financial time series to investigate the extent of interdependence between different assets. In some cases, for example [19], the dynamics of higher order moments is considered as a measure of the level of risk being shared among asset classes. In [19], the price dynamics are considered to elucidate the dependence of the higher order moments of different assets for the carbon and energy markets within the EU ETS. As can be seen, to accurately represent complex phenomena within financial settings, a proper description and understanding of the higher order moments can be instrumental.

It has been stated by many researchers, [20], that the number of rare-events is inextricably linked to the level of risk within a process. This indicates that we can use the higher order moment analysis as a tool to indicate changes of a financial asset's risk. It has been shown that a very effective methodology for measuring risk is Value-at-Risk (VaR), [21]. Therefore, we will use VaR to look at the link between the higher order moments and the risk within the financial data series.

The paper is organised as follows; in section 2.1, we introduce the scaling relations in ($\Gamma_{4}$, $\Gamma_{6}$) space. In section 2.2, we consider the economic periods we wish to analyse whilst presenting the results for the empirical data. In Section 2.3, we compare the higher order standardised moments obtained from the empirical data with the corresponding moments for the gaussian distribution. Section 3 shows that scaling relations are linked to the hierarchy of rare events and the exponent of price power law distributions. In addition, we develop an approach linking the obtained scaling with stock risk management inspired by previous studies, [20], [21], [22], [23], [24], [25], where higher order moments were used for risk assessment. In section 5, we highlight that the change in the dependence of the Value-at-Risk on the time window duration occurs at the same values when we observe changes of the scaling relations of higher order moments. Finally, section 6 concludes.

## 2 Empirical data processing

### 2.1 Estimation of higher order moments from empirical data

Throughout the paper, we will consider time windows of *N* trading days and analyse the logarithm of stock returns defined as:

(1) $$x_{i}=\ln\left(\frac{y(t_{0}+i\delta t)}{y(t_{0}+(i-1)\delta t)}\right)$$

where $t_{0}$ being the date of the first trading day within the studied window, $y(t_{0}+(i-1)\delta t)$ is the closing price on the *i*th trading day with 1≤i≤N and *δt* referring to the time between trading days. Using the logarithm of stock returns, we estimate the nth order standardised moments:

(2) $$\Gamma_{n}(t_{0},N)=\frac{\langle {\left(x-\mu \right)}^{n}\rangle }{{\langle {\left(x-\mu \right)}^{2}\rangle }^{\frac{n}{2}}}$$

where,

(3) $$\langle {\left(x-\mu \right)}^{n}\rangle =\frac{1}{N}\sum_{i=1}^{N}{\left(x_{i}-\mu \right)}^{n}$$

and,

(4) $$\mu =\frac{1}{N}\sum_{i=1}^{N}x_{i}.$$

We analyse the dependence of $\Gamma_{n}(t_{0},N)$ on both number of trading days *N* in the window and the absolute time $t_{0}$ with a goal to observe and analyse empirical laws and their evolution during the economic crisis. In addition, we compare $\Gamma_{n}(t_{0},N)$ for different *N* and n=2,4,6,8,10,12 with the corresponding values of gaussian standardised moments.

**Figure 1:**
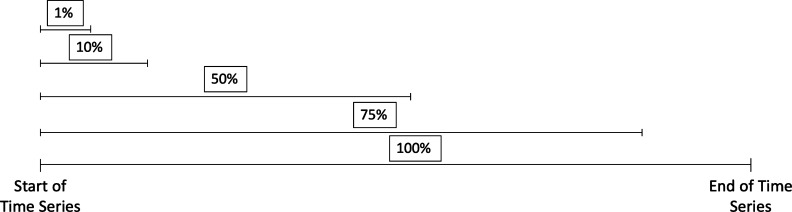
The truncation method for a time series within this paper. We take a long time series, labelled here as 100%. We start by taking 1% of its length and increment up by 0.1% to its original length, 100%. For the analysis we undertake here, the longest time series is 18-years. In this figure, we label the 1%, 10%, 50% and 75% time lengths to show how the time series lengths increases.

To investigate the scaling relations of higher order standardised moments, we truncate an 18 year (6th October 2000 to 6th October 2018) time series. To do this, we take the long time series and segment it to a length of 1% of the original time window's length. We then gradually increase the time window by 0.1% of the original time series' length up to 100% of the whole time series (18 years). This corresponds to around 4536 days. We therefore will gain 1000 time series out of the original 18 year time series. The schematics of this truncation method can be seen in Fig. 1.

By calculating the *X*-th and *Y*-th order statistical moments of the empirical data in all 1000 windows (see the method described above), we are able to present the data in ($\Gamma_{X}$, $\Gamma_{Y}$) space. We will use different orders, *X* and *Y*, of the standardised moments and search for empirical relationships between them. For example, for relations between the fourth and sixth order moments, we will use Y=6 and X=4. To analyse the behaviour of the market data in response to the truncation of the time series we propose to use scaling relations:

(5) $$\Gamma_{Y}(t_{0},N)=A\Gamma_{X}{\left[t_{0},N\right]}^{B}$$

where A and B are constants. In logarithmic scale this reduces to a straight line:

(6) $$\ln(\Gamma_{Y})=B\ln(\Gamma_{X})+\ln(A).$$

Below, we will refer to the parameter *B* as either the scaling exponent or the logarithmic gradient.

**Figure 2:**
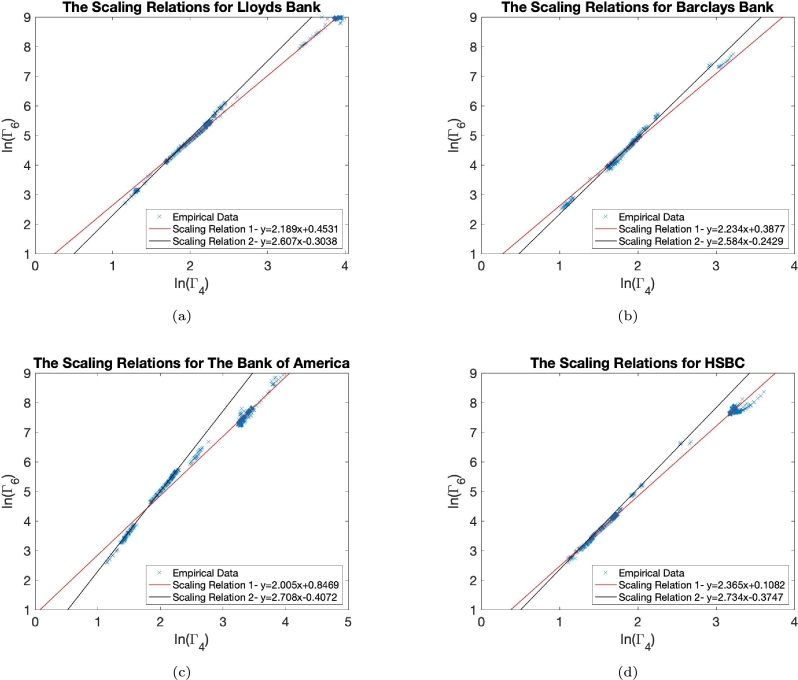
Here, we show the data points on the ($\ln(\Gamma_{4})$, $\ln(\Gamma_{6})$) phase space and the two scaling relations discussed in the text. The black line shows the scaling relation for the shorter time windows, whilst the red line shows the scaling relation for the longer time windows. In panel (a), we show Lloyds Bank, (b), Barclays Bank, (c), Bank of America and (d), HSBC.

Fig. 2 uncovers two different scalings for four banking companies. The first relation, shown in red for all companies, is the scaling relations for the longer time windows. However, we discover a different scaling behaviour with different exponents *B* for shorter time windows. Such a distinct two scaling behaviour has been observed for all companies studied. We also observe similar scaling relations for other standardised moments. For example, in Appendix B, we present the scaling relations for the fourth $\Gamma_{4}$ and eighth $\Gamma_{8}$, as well as the fourth, $\Gamma_{4}$ and tenth $\Gamma_{10}$. Since the odd moments can change sign, we can analyse if they still exhibit the proposed power law (5) dependence only within the interval where the odd order moments do not change sign and by calculating their absolute value if necessary. This significantly restricts the number of considered time windows. For example, in Appendix B we present our analysis for the odd order standardised moments, $\Gamma_{3}$ and $\Gamma_{5}$, but only for time windows greater than 2000 events. Extending such analysis to odd order moments, we also see the double scaling relations evident for the empirical data (see below).

### 2.2 The impact of economic environments upon scaling relations

**Figure 3:**
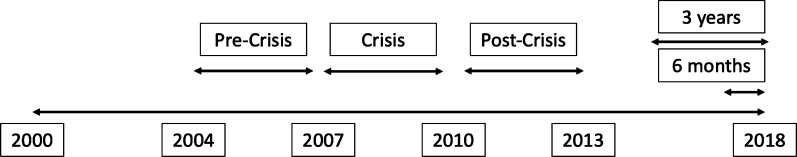
The timeline of time series used for the three periods of economic cycles and the truncation data, [10].

We now turn our attention to the effect of the economic environment on the scaling relations. To investigate this we use the economic periods set out in Fig. 3. Here, we have a pre-crisis period, 2005, before the financial crash, a crisis period, 2008 and then the post-crisis period, 2011. For completeness, we investigate the succeeding years of 2014 and 2017, to see the effect the financial crisis has upon the scaling relations over a prolonged period of time.

The scaling relations for these periods are worked out using the same method as described above for a 252 day time series, however, we do not consider time windows shorter than 25 days, that is we ignore time windows with very small statistics. The results can be found in Table 1, where Y is the $ln(\Gamma_{6})$ and X is the $\ln(\Gamma_{4})$ for the longer window scaling. It is clear from the scaling relations found, the economic period has a very vivid effect upon the companies behaviour.

**Table 1:** Here, we present the scaling relations for the longer time horizons for several companies of different economic environments. Y, represents the logarithm of the sixth order standardised moment and X the logarithm of the fourth order standardised moment, with the coefficient in front of X being the logarithmic gradient, *B*.

Company	2005	2008	2011	2014	2017
Barclays	Y=7.4X−9.8	Y=31.3X−112.4	Y=12.1X−25.4	Y=12.6X−23.8	Y=8.3X−12.4
Bank Of America	Y=6.7X−8.5	Y=18.7X−45.4	Y=24.8X−66.4	Y=9.6X−14.5	Y=15.7X−29.8
Gold	Y=9.3X−15	Y=28.3X−87.2	Y=10.5X−15.9	Y=23.4X−68.4	Y=12.4X−25
GSK	Y=6.4X−3	Y=11.7X−20.2	Y=13.2X−25.8	Y=33X−70.6	Y=22.12X−49.9
Lloyds	Y=21.2X−44	Y=35.2X−172.9	Y=12.3X−23.2	Y=11.4X−18.2	Y=8.9X−11
Rio Tinto	Y=9.8X−14.8	Y=14X−27.1	Y=10.7X−19	Y=9.3X−14.6	Y=11X−17

For instance, if we analyse Lloyds Bank. The scaling relation for the pre-crisis period, has a logarithmic gradient B=21.2, whereas, within and just after the crisis period the exponent, *B* increases drastically. After the crisis period, the exponent, *B*, decreases to a lower level than the pre-crisis period. This indicates, the long term impact of the financial crash. As we do not see this behaviour of the exponent in other security types, we can infer that this behaviour is due to these companies being directly affected by the financial crash of 2008. The fact we have persistence of this effect can be seen as an indication that the financial crisis period has long run dynamical impacts upon the market price of these companies. The same behaviour, however, can be seen for the Gold scaling relations. We see an increase of the exponent, *B*, in response to the financial crisis, followed by the exponent returning to a level similar to the pre-crisis environment. However, the striking increase of logarithmic gradient, *B*, followed by its post-crisis drop observed for banking companies, has not been found in non-banking sectors of the economy. For example, GSK (a pharmaceutical company) and Rio Tinto (a metals and mining corporation) do not have such a distinguished behaviour. In the case of Rio Tinto, the gradient stays relatively static throughout the time, whereas, GSK has an increase in 2014, which could be attributed to the bribery scandal that encompassed the company from 2013 to 2014, [26]. It can therefore be said that the financial crisis has more of an effect upon the banking companies, as we would expect due to the nature of the cause of the crisis period.

### 2.3 Higher order standardised moments in empirical data

**Figure 4:**
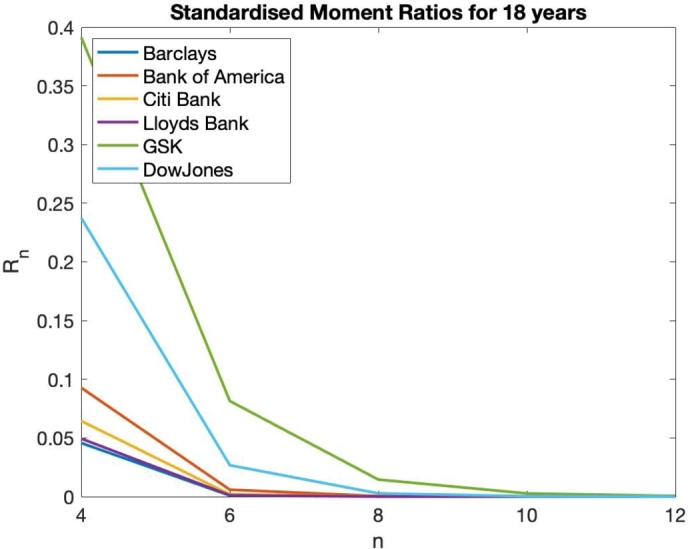
The standardised moment ratios for varying orders, *n*, see Appendix A, for the gaussian standardised moments divided by the empirical values. Here, we have analysed the 18 year time series for Bank of America, Barclays Bank, Citi Bank, the DowJones Index, GSK, HSBC and Lloyds Bank.

Here, we compare the higher order standardised moments defined by equation (2) for *n*=4, 6, 8, 10 and 12 and compare them with the corresponding gaussian standardised moment values listed in Fig. 4. The equation for this ratio, $R_{n}$, is shown below:

(7) $$R_{n}=\frac{\Gamma_{n}^{gaussian}}{\Gamma_{n}}.$$

**Figure 5:**
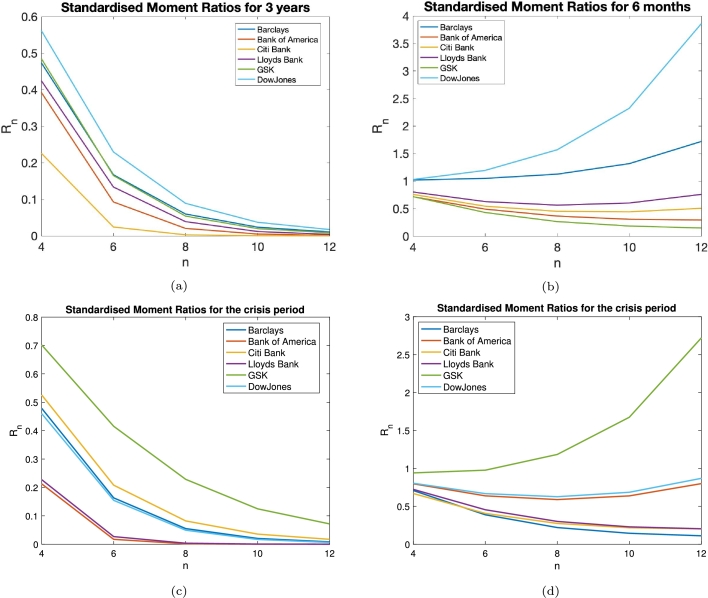
The ratios for the gaussian higher order standardised moments to the empirical ones. Here we have analysed, the 3 year, panel (a), and the 6 month, panel (b), time series for the Bank of America, Barclays Bank, Citi Bank, the DowJones Index, GSK and Lloyds Bank. We consider time windows from 6th October 2015-6th October 2018, panel (a), and 6th April 2018-6th October 2018, panel (b). Remarkably, for some companies' 6 month time series the ratio of gaussian to empirical standardised moments is above one in contrast to results shown in Fig. 4 and 5a. In addition, Lloyds Bank (purple curve) shows a non-monotonic behaviour as standardised moment order increases. Panel (c), shows the same for the crisis period (2007-2009, inclusive). We see a decay with respect to the increasing *n*. Panel (d), shows the higher order standardised moment ratios for the 6 month time series from the start of the financial crisis, 1st January 2007-1st July 2007.

The results for various companies and their market data can be seen in Fig. 4. We also show ratios (7), for shorter time windows, namely, 3 years and 6 months. The reason for the use of a 3 year time window is to isolate the crisis period (2007-2009), which is around 3 years. Therefore, in taking a 3 year window, we are able to stop leakage of the effects of a crisis period into the pre- and post-crisis periods. This helps us to investigate the nature of these periods without mutual interference. Moreover, we select the short 6 month period due to the reliability of statistics. 6 months, represents around 126 events. We note that for a gaussian statistics, the error scales as 1/n where *n* is the number of observations, [27]. Therefore, the statistical error for 6 month time window is about 1%. The shorter time window can result in higher fluctuations, justifying our choice. The results of which can be seen in Fig. 5. The values of the higher order standardised moments of the empirical financial series for the 3 years time window can be found in table A.4, in Appendix A.

The evolution of higher order moments of the empirical financial series is quite remarkable. When we take a long time series, either the 18 years or the 3 years (Figs. 4 or 5a), the ratio of gaussian to empirical standardised moment is below 1, which we can expect for leptokurtic distributions. When we instead truncate this time series to 6 months, Fig. 5b, we get some empirical higher order standardised moments that are now less than that of the gaussian values. Moreover, we uncover the decay of the ratios as a function of its order for long series (18 and 3 years) which unexpectedly start to grow or even have a non-monotonic behaviour for shorter time windows (6 months). Comparing the crisis and post-crisis period behaviour, we have observed that depending on the economic environment, different companies exhibit intriguing non-monotonic behaviour shown. In the crisis period (Fig. 5d), the DowJones and the Bank of America time series exhibit such behaviours, whereas, for the post-crisis period, it is the Lloyds Bank time series that shows such a feature. A possible reason for this behaviour in the DowJones and the Bank of America may be an elucidation to the financial crisis, whilst in Lloyds Bank could be attributed to the fact that the Government sold its remaining stake in the bank close to this period.

## 3 GARCH simulations

**Figure 6:**
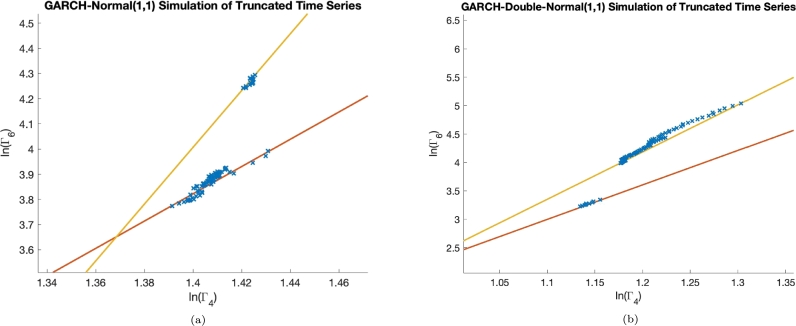
In panel (a), we show the simulation results for a GARCH-normal(1,1) model, when we take the parameter values; *α*_0_ = 1*e* − 5, *α*_1_ = *β*_1_ = 0.3. We see two distinct scaling relations with respect to the sample size. We see one scaling relation when we take longer time windows, shown by the yellow line, with equation; Y=11.3X−11.79, and another when we take smaller time windows, shown in red, with an equation Y=5.42X−3.77. In panel (b), we show the simulation results for a GARCH-double-normal(1,1) model. Here, we see a scaling relation for the shorter time windows, shown by the red line, given by the equation, Y=6.07X−3.68 and one for the longer time windows, shown by the yellow line, given by the equation, Y=8.31X−5.79.

To understand the origins of these different scaling relations we simulate a GARCH-normal(1,1) model and a GARCH-double-normal(1,1) model, used before in, [10], [28], [29]. We first simulate a GARCH model with a given conditional distribution and a given set of time-independent parameters for a large number of events. Once this has been done, we then truncate the long time series into subsets of the whole time series. This truncation is the same as described in section 2.1. For each time series, we calculate the fourth and sixth order standardised moments and plot $\ln(\Gamma_{6})$ versus $\ln(\Gamma_{4})$, see Fig. 6. In order to simulate events, the dynamic equation for $\sigma_{t}^{2}$, the conditional variance, is used:

(8) $$\sigma_{t}^{2}=\alpha_{0}+\alpha_{1}x_{t-1}^{2}+\beta_{1}\sigma_{t-1}^{2}$$

where, $x_{t}=\chi_{t}\sigma_{t}$, and $\chi_{t}$ is an independent identically distributed random variable with standard deviation equal to 1 and a mean of zero. For the Normal model, $\chi_{t}$ is described by a gaussian distribution. We use the following parameter values for the GARCH-normal(1,1) model; $\alpha_{0}=1\times 10^{-5}$, $\alpha_{1}=0.3$ and $\beta_{1}=0.3$. In Fig. 6a, we see the scaling laws for the GARCH-normal(1,1) model. We can see one scaling relation pertain to smaller time windows, shown in red with an equation Y=5.42X−3.77 and another for longer time windows, shown in yellow with an equation, Y=11.28X−11.79. We only see two distinct scaling relations for a very limited number of parameter choices. When we instead use a GARCH-double-normal(1,1) model we recover the two scaling relations in a much wider parameter region, which results in a better fit to the scaling laws. For the double normal GARCH model, the distribution of $\chi_{t}$ has the form:

(9) $$p(x)=\frac{a}{\sigma_{1}\sqrt{2\pi }}e^{-x^{2}/(2\sigma_{1}^{2})}+\frac{b}{\sigma_{2}\sqrt{2\pi }}e^{-x^{2}/(2\sigma_{2}^{2})}$$

We assign the following values for the parameters of the distribution (9); a=0.9818, b=0.0182, $\sigma_{1}^{2}=0.833$ and $\sigma_{2}^{2}=9.986$. We simulate, a return time series and truncate the series in the exact manner that has been undertaken for the empirical data above. To do this, we use the following GARCH parameter values for the double gaussian model; $\alpha_{0}=1e-5$, $\alpha_{1}=0.5$ and $\beta_{1}=0$.

The results for the GARCH simulation of studied scaling laws can be seen in Fig. 6b. Here, we see the two scaling relations for the different time scales. For the shorter time windows (the red line), we have a straight line equation of; Y=6.07X−3.68 and for the longer time horizons (the yellow line), we have the equation; Y=8.31X−5.79. There is a clear difference in the scaling relations of the simulated data and the empirical data, namely in the simulated data the logarithmic gradient, *B*, for the shorter time window is lower than the longer time window. Something that is not mirrored in the empirical data. Now, if we change the parameters $\alpha_{1}$ and $\beta_{1}$ we will be able to highlight how the dependence on the past level of return and past level of volatility impacts the scaling relations. If we increase $\beta_{1}$, to 0.8 and reduce $\alpha_{1}$ to 0.1, we see that the longer time horizon scaling relation is still steeper than the shorter one. The same is true when we increase $\alpha_{1}$ to 0.6 and reduce $\beta_{1}$ to 0.1. However, we do see a connection of the value of $\alpha_{1}$ to the value of the logarithmic gradient for the scaling relations. When we increase $\alpha_{1}$, the logarithmic gradient, *B*, also increases. That is to say, the more of a dependence the past return has on the future volatility level, the larger the value of the logarithmic gradient, *B*.

## 4 Hierarchical analysis of rare-events

In order to analyse the behaviour of the higher order moments of the logarithm of price returns in the truncated time windows having *N* trading days, we note that the rare-events whose probability is extremely low, will not contribute to the higher order moment calculation within this window. Indeed, the probability to observe tradings with returns $|x|>x_{W}$ occurring within *N* days can be evaluated as $P_{N}(x_{W})=2N\int_{x_{W}}^{\infty }p(x)dx$ with a probability distribution, p(x), of the logarithm of price returns (for simplicity we assume p(x) to be an even function). If P≪1, we can safely ignore such events and evaluate the higher order moments within interval $|x|<x_{W}$, where $x_{W}$ can be estimated from the condition that $P_{N}(x_{W})=C∼1$, when *C* is a constant. This allows us to evaluate the higher order moments for a *N*-days trading window, using the following equations:

(10) $$2N\int_{x_{W}}^{\infty }p(x)dx=C\langle x^{n}\rangle =2\int_{0}^{x_{W}}x^{n}p(x)dx$$

The empirically observed two distinct scaling laws suggest that the probability distribution should have two different functional behaviours at large *x* resulting in a hierarchy of rare-events in two groups: rare-events and very rare-events. This can be done using a usual Paerto tail distribution whose exponent changes from $\gamma_{1}$ to $\gamma_{2}$ at certain $|x|=x_{1}$:

(11) $$p(x)=\left\{ \begin{array}{cc} 0 & \text{if }|x|<x_{0}\\ A{\left|\frac{x}{x_{1}}\right|}^{-\gamma_{1}} & \text{if }x_{0}<|x|<x_{1}\\ A{\left|\frac{x}{x_{1}}\right|}^{-\gamma_{2}} & \text{if }|x|>x_{1}. \end{array}\right.$$

Note that our analysis below does not depend on the behaviour of a probability density at small values of |x|, thus we assume that p(x)=0 for $|x|<x_{0}$, for simplicity of our estimations. Substituting equation (11) into the set of equations (10) and restricting our analysis to $3<\gamma_{1}<5$ and $3<\gamma_{2}<5$, we derive the short-window scaling relations for $x_{0}\ll x_{W}\ll x_{1}$:

(12) $$x_{W}=R_{1}N^{\frac{1}{1-\gamma_{1}}},\Gamma_{4}=K_{4}{\left(x_{W}\right)}^{5-\gamma_{1}},\Gamma_{6}=K_{6}{\left(x_{W}\right)}^{7-\gamma_{1}}$$

and the long-window scaling relations for $x_{1}\ll x_{W}$:

(13) $$x_{W}=R_{2}N^{\frac{1}{1-\gamma_{2}}}\;\Gamma_{4}=Q_{4}{\left(x_{W}\right)}^{5-\gamma_{2}},\Gamma_{6}=Q_{6}{\left(x_{W}\right)}^{7-\gamma_{2}}$$

where $R_{1},R_{2},K_{4},K_{6},Q_{4},Q_{6}$ do not depend on *N*. When deriving the above equation we keep only the main contributions to the integrals, for example, approximating ${\left(x_{0}\right)}^{3-\gamma_{1}}-{\left(x_{W}\right)}^{3-\gamma_{1}}\approx {\left(x_{0}\right)}^{3-\gamma_{1}}$ and ${\left(x_{W}\right)}^{5-\gamma_{1}}-{\left(x_{0}\right)}^{5-\gamma_{1}}\approx {\left(x_{W}\right)}^{5-\gamma_{1}}$.

From the above set of equations we derive two different scaling laws, these laws have been observed in the empirical data:

(14) $$\Gamma_{6}=L_{1}\Gamma_{4}^{\frac{7-\gamma_{1}}{5-\gamma_{1}}}$$

which is valid for short time windows $N\ll {\left(x_{1}/R_{1}\right)}^{\gamma_{1}-1}$, and:

(15) $$\Gamma_{6}=L_{2}\Gamma_{4}^{\frac{7-\gamma_{2}}{5-\gamma_{2}}},$$

which is valid for long time windows, $N\gg {\left(x_{1}/R_{2}\right)}^{\gamma_{2}-1}$. Where, $L_{1}$ and $L_{2}$ are constants. In order to reproduce the empirical observation that the shorter time windows have a steeper gradient in the ($ln(\Gamma_{4})$, $ln(\Gamma_{6})$) space we have to request that $\gamma_{1}>\gamma_{2}$. This means, the steeper the gradient in this higher order moment space, equates to a faster decay in the probability distribution with respect to price change, *x*. Using this analysis, we are able to see that depending where we take this truncation, $x_{W}$, we will potentially expose ourselves to a higher level of risk to these rare-events. This is due to the truncation determining the forecasting horizon we are interested in. Therefore, we can see that the length of window we wish to model will have an effect on the risk level we expose ourselves to during this period. In order to hedge for a higher level of risk than we may expect, we must ascertain which exponent of the power law the distribution has for events occurring with frequency 1/N, when forecasting a *N* trading day horizon. In doing so, we can correctly calculate the risk we are exposing our position to.

## 5 Implications on value-at-risk

**Figure 7:**
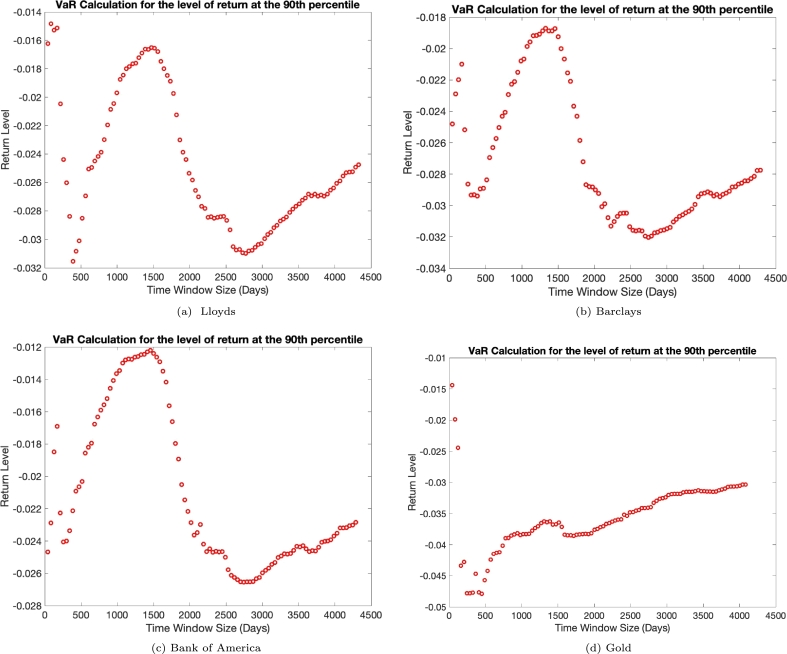
The Value-at-Risk Calculations for the 90th confidence level of returns for Lloyds, panel (a), Barclays, panel (b), Bank of America, panel (c) and Gold ETFs, panel (d). To construct such diagrams, we truncate the empirical data to 1% of its original length and increment in 0.1% up to its total length. We then work out the 90th confidence level, see text for details of the VaR calculation. We would expect to see a consistent increase in the level of loss of return in response to the increased time window. However, this is not seen and instead we see two distinct behaviours with regards to the level of loss. One region where we get sensical behaviour, increase losses for an increased time window and a second, where we get a decrease in the level of loss for the increased time window.

To deduce the implications of these different scaling relations on the level of risk, we use a simple Value at Risk (VaR) calculation, [30], [31]. The aim of using this simple risk calculation is to demonstrate the effect rare-events have upon the level of risk within a return series. We have seen from preceding sections, the scaling laws are apparent in all of the empirical time series we have analysed in this paper. It is well documented, [20], that the quantity of rare-events within a process affects the risk associated with such a process. It is therefore sensible to assume the scaling relations we have seen, should manifest themselves in a risk calculation of the same financial time series. To calculate this risk, we shall carry out a very simple Value-at-Risk routine. To do so, we take the return series for the time window we are investigating and order the returns into descending order, we then look to find the smallest 10% of the returns, which equate to the top 10% of losses. Therefore, we will analyse the level of loss that can be expected at the 90% confidence interval for the corresponding time window. In Fig. 7, we see the level of return for the 90th confidence level for the truncated time windows for Lloyds Bank, Barclays bank, Bank of America and Gold ETFs.

In this figure, we can see some distinct regions in the level of risk. For the shortest time window, we have a relatively low level of loss, which is to be expected given the short time window. This equates to less uncertainty. When we increase the time window we get an increase in the loss, as we would expect. This consequently, is a result of the increase in the uncertainty in the return level given more data. However, starting from a certain point the increase stops and instead reverses. Now, we have a decreasing level of loss for an increasing time window. This is the point at which we gain a different scaling relation in the ($\ln(\Gamma_{4})$, $\ln(\Gamma_{6})$) phase space. However, there is now a disparity between the banking securities and gold. Whilst, the banking securities continue to decrease the level of loss with the time window, gold reverses again and starts to increase the loss for the increasing time window. For the banking securities, we get a much longer decrease with respect to the time window length. However, we do see this region end around the 200th data point and instead, we get a third regime where the level of loss starts to increase with increased length of time window, a behaviour we would expect.

## 6 Conclusion

By the use of higher order moments, we uncover a new scaling behaviour of the empirical data. For the longer time windows, the logarithm of the fourth and sixth order standardised moments follows a straight line. The same behaviour was observed for the shorter time windows but with different parameters of the scaling equation. This fact is seen throughout all of the empirical data we have analysed for different financial data series.

We also highlight the impact of differing economic periods upon these scaling relations via the investigation of the empirical data throughout the 2008 financial crash. Here, we show that for companies directly affected by the crash, primarily banking companies, there is a drastic change to the logarithmic gradient of the scaling relation. This impact is long lasting in the empirical data. Almost a decade after the crash there is still an evident legacy of this economic period in the empirical data's higher order moments.

Furthering the investigation of empirical data, we show the relationship between the higher order standardised moments of the empirical data and the standardised moment values of the gaussian distribution. We show by truncating the data into 18 years, 3 years and a 6 month time series, the length of time we investigate over has a stark impact on the higher order standardised moments.

Moreover, we try to replicate the observed scaling laws using a GARCH(1,1) models. We are able to show that for all parameter values investigated, we gain two distinct scaling relations. However, we get the longer time window's scaling relation to have a larger logarithmic gradient, *B*, than the shorter one. A clear contradiction to the empirical data. We resolve this rather puzzling behaviour by modelling rare-events in different time windows.

In order to deduce the behaviour of risk we carry out a Value-at-Risk type calculation to determine the potential loss at the 90th confidence level for the different time horizons analysed. We see that for the different scaling relations we encounter different levels of risk. We would expect that for an increasing time horizon, the risk increases, however, when we have the observed change in scaling relation of the standardised higher order moments we encounter a reduction in the level of loss for an increase in time horizon. In conclusion, the data analysis reported in this paper can elucidate the behaviour of prices within short and long time horizons and as such can be used as a useful tool for market and portfolio analysis. The above described empirical data analysis provides a way of how financial data can be used to train neuromorphic hardware, [32].

## Declarations

### Author contribution statement

Luke De Clerk: Conceived and designed the experiments; Performed the experiments; Analyzed and interpreted the data; Contributed reagents, materials, analysis tools or data; Wrote the paper. Sergey Savel'ev: Conceived and designed the experiments; Analyzed and interpreted the data; Contributed reagents, materials, analysis tools or data; Wrote the paper.

### Funding statement

This work was supported in part by the 10.13039/501100000266Engineering and Physical Sciences Research Council (EPSRC) (Grant No. EP/S032843/1).

### Data availability statement

Data will be made available on request.

### Declaration of interests statement

The authors declare no conflict of interest.

### Additional information

Supplementary material related to this article can be found online at https://doi.org/10.1016/j.heliyon.2022.e08833.

No additional information is available for this paper.

## Supplementary material

appendix_supp_mat.pdf Here, we detail the tables of data for the 3-year and 18-year higher order standardised moment ratios, as well as the values for the gaussian distribution. Additionally, we enclose the figures for the scaling relations in the fourth and the eighth or tenth order standardised moment space for the time series of Lloyds Bank and Bank of America. Lastly, we present the scaling relations for Lloyds Bank in the third and fourth order standardised moment space, the fourth and fifth order standardised moment space and the fourth and sixth order standardised moment space for the filtered time window. appendix_supp_mat.pdf

## References

[1] Ding Z., Engle R., Granger C. (February 1993). A long memory property of stock market return and a new model. J. Empir. Finance.

[2] Weide R. (October 2002). A multivariate generalised orthogonal garch model. J. Appl. Econom..

[3] Breen W., Glosten L., Jagannathan R. (1989). Economic significance of predictable variations in stock index returns. J. Finance.

[4] Nelson D. (March 1991). Conditional heteroskedastcicity in asset returns: a new approach. Econometrica.

[5] Engle R., Gallo G. (April 2006). A multiple indicators model for volatility using intra-day data. J. Econom..

[6] Duan J., Gauthier G., Simonato J., Sasseville C. (April 2006). Approximating the gjr-garch and egarch option pricing models analytically. J. Comput. Finance.

[7] Black F., Scholes M. (May 1973). The pricing of options and corporate liabilities. J. Polit. Econ..

[8] Chan K., Karolyi G., Stulz R. (1992). Global financial markets and the risk premium on U.S. equity. J. Financ. Econ..

[9] Merton R.C. (April 1973). Theory of rational option pricing. J. Econom..

[10] Clerk L.D., Savel'ev S. (February 2021). Non-stationary modelling of garch to fit higher order moments of financial series within fixed time windows. arxiv:2102.11627.

[11] Mantegna R., Stanley H. (2000).

[12] Lim G.C., Martin G.M., Martin V.L. (2005). Parametric pricing of higher order moments in sp500 options. J. Appl. Econom..

[13] Xu W., Wu C., Li H. (2011). Accounting for the impact of higher order moments in foreign equity option pricing model. Econ. Model..

[14] Doan M.P., Lin C.-T. (2012). On the robustness of higher-moment factors in explaining average expected returns: evidence from Australia. Res. Int. Bus. Finance.

[15] Tanaka K., Yamada T., Watanabe T. (2010). Applications of Gram-Charlier expansion and bond moments for pricing of interest rates and credit risk. Quant. Finance.

[16] Jurczenko E., Maillet B., Negrea B. (2004). A note on skewness and kurtosis adjusted option pricing models under the martingale restriction. Quant. Finance.

[17] Dhesi G., Shakeel B., Ausloos M. (2021). Modelling and forecasting the kurtosis and returns distributions of financial markets: irrational fractional Brownian motion model approach. Ann. Oper. Res..

[18] Scott R., Horvath P. (1980). On the direction of preference for moments of higher orders than the variance. J. Finance.

[19] Dai X., Xiao L., Wang Q., Dhesi G. (2021). Multi-scale interplay of higher order moments between the carbon and energy markets during phase 3 of the eu ets. Energy Policy.

[20] Longin F. (2000). From value at risk to stress testing: the extreme value approach. J. Bank. Finance.

[21] Huang Y., Dai X., Wang Q., Zhou D. (2021). A hybrid model for carbon price forecasting using garch and long short-term memory network. Appl. Energy.

[22] Sihem M., Slaheddine H. (2014). The impact of higher order moments on market risk assessment. Proc. Econ. Finance.

[23] Perez-Quiros G., Timmerman A. (2001). Business cycle asymmetries in stock returns: evidence of higher order moments and conditional densities. J. Econom..

[24] Harvey C.R., Liechty J.C., Liechty M.W., Müller P. (2010). Portfolio selection with higher order moments. Quant. Finance.

[25] Javed F., Mazur S., Ngalio E. (2021). Higher order moments of the estimated tangency portfolio weights. J. Appl. Stat..

[26] Neate R. Glaxosmithkline ‘the big boss’ in £300m bribery scandal, China says. https://www.theguardian.com/business/2013/jul/15/glaxosmithkline-china-bribery-allegations.

[27] Tsay R. (2010).

[28] Haas M., Krause J., Paolella M.S. (2013). Time-varying mixture GARCH models and asymmetric volatility. N. Am. J. Econ. Finance.

[29] Alexander C., Lazar E. (2006). Normal mixture garch(1,1): applications to exchange rate modelling. J. Appl. Econom..

[30] Bali T.G., Theodossiou P. (2007). A conditional sgt-var approach with alternative garch models. Ann. Oper. Res..

[31] Orhan M., Köksal B. (2021). A comparison of garch models for var estimation. Expert Syst. Appl..

[32] Wang Z., Joshi S., Savel'ev S., Song W., Midya R., Li Y., Rao M., Yan P., Asapu S., Zhou Y., Jiang H., Lin P., Li C., Yoon J.H., Upadhyay N.K., Zhang J., Hu M., Strachan J.P., Barnell M., Wu Q., Wu H., Williams R.S., Xia Q., Yang J.J. (2018). Fully memristive neural networks for pattern classification with unsupervised learning. Nat. Electron..

