# Supplementary material for: An investigation of higher order moments of empirical financial data and their implications to risk
Source: Heliyon. 2022 Feb 3;8(2):e08833. doi: 10.1016/j.heliyon.2022.e08833 (PMC8841368; doi:10.1016/j.heliyon.2022.e08833)
Supplement: appendix_supp_mat.pdf — Here, we detail the tables of data for the 3-year and 18-year higher order standardised moment ratios, as well as the values for the gaussian distribution. Additionally, we enclose the figures for the scaling relations in the fourth and the eighth or tenth order standardised moment space for the time series of Lloyds Bank and Bank of America. Lastly, we present the scaling relations for Lloyds Bank in the third and fourth order standardised moment space, the fourth and fifth order standardised moment space and the fourth and sixth order standardised moment space for the filtered time window. [file mmc1.pdf]

Appendix A. Standardised Moments Order and Gaussian Values

Calculation Number	Equation	Gaussian Value, $\Gamma_n^{gaussian}$
A	$\frac{\langle (x-\mu)^4 \rangle}{\langle (x-\mu)^2 \rangle^2}$	3
B	$\frac{\langle (x-\mu)^6 \rangle}{\langle (x-\mu)^2 \rangle^3}$	15
C	$\frac{\langle (x-\mu)^8 \rangle}{\langle (x-\mu)^2 \rangle^4}$	105
D	$\frac{\langle (x-\mu)^{10} \rangle}{\langle (x-\mu)^2 \rangle^5}$	945
E	$\frac{\langle (x-\mu)^{12} \rangle}{\langle (x-\mu)^2 \rangle^6}$	10395

Table A.1: The equations of the standardised moment calculations for those used in figure **??**. We also display the corresponding gaussian values.

Standardised Moment Calculation Number	Bank of America	Barclays Bank	Citigroup	HSBC	Lloyds Bank
A	16.51	46.47	7.14	6.07	26.02
B	1.46e03	1.18e04	124.17	98.55	2.58e03
C	1.78e05	3.37e06	3.12e03	2.65e03	3.22e05
D	2.30e07	9.65e08	9.32e04	9.15e04	4.35e07
E	3.04e09	2.77e11	3.05e06	3.56e06	6.16e09

Table A.2: The values of the raw data standardised moments for the 18 year time series for the companies used in figures **??**, **??**. The ordering of the standardised moment calculations are the same as those seen in table **??**.

Standardised Moment Calculation	Barclays	BoA	Citi	Lloyds	GSK	DowJones
A	4.4013	4.9296	4.1463	3.369	6.1169	8.6692
B	36.017	41.762	29.324	17.696	79.605	149.98
C	389.98	449.33	264.94	114.97	1391.8	3274.5
D	4864.9	5442.7	2730.9	845.15	27453	79051
E	65468	69803	30472	6733.8	5.69E+05	2.00E+06

Table A.3: The values of the raw data of the standardised moment calculations for the three year time series of the companies used in figure **??**, specifically figure **??**, the ordering of the values is the same as that shown in table **??**.

Appendix B. Higher order standardised moment scaling relations

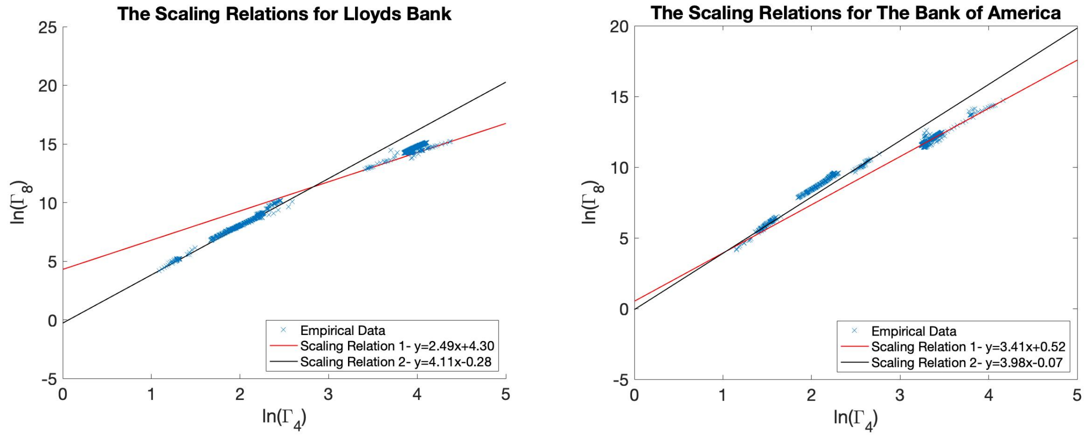

(a) (b)

Figure B.1: Here, we present the scaling relations in for the fourth and eighth order standardised moments. In panel (a), we show the relation for Lloyds Bank, with the shorter scaling relation being, $\mathcal{Y} = 4.11\mathcal{X} - 0.28$, in black and the longer relation, $\mathcal{Y} = 2.49\mathcal{X} + 4.30$, in red. In panel (b), we see the scaling relations for Bank of America, $\mathcal{Y} = 3.98\mathcal{X} - 0.07$, in black, whilst the longer time windows, $\mathcal{Y} = 3.41\mathcal{X} + 0.52$, in red.

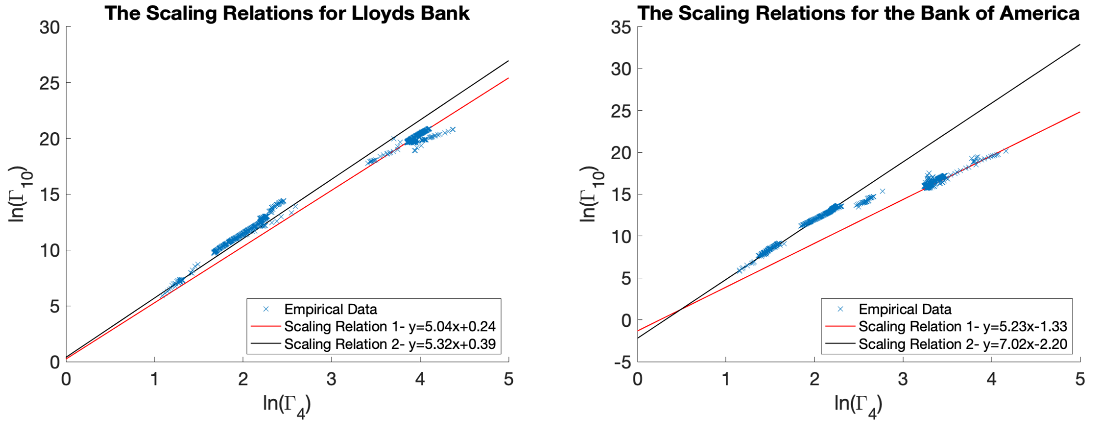

(a) (b)

Figure B.2: Here, we present the scaling relations for the fourth and tenth order standardised moments. In panel (a), we show the relation for Lloyds Bank, with the shorter scaling relation being, $\mathcal{Y} = 5.32\mathcal{X} + 0.39$, in black and the longer relation, $\mathcal{Y} = 5.04\mathcal{X} + 0.24$, in red. In panel (b), we plot the scaling relations for Bank of America, the scaling relation is, $\mathcal{Y} = 7.02\mathcal{X} - 2.20$, in black, $\mathcal{Y} = 5.23\mathcal{X} - 1.33$, in red.

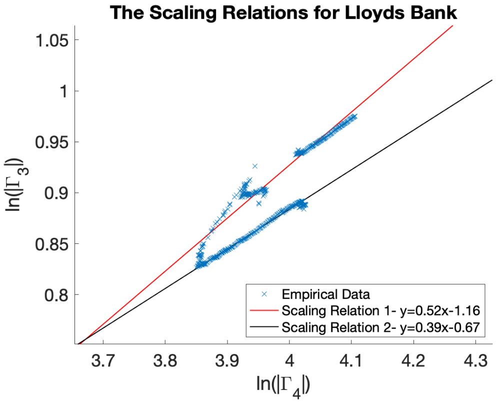

(a)

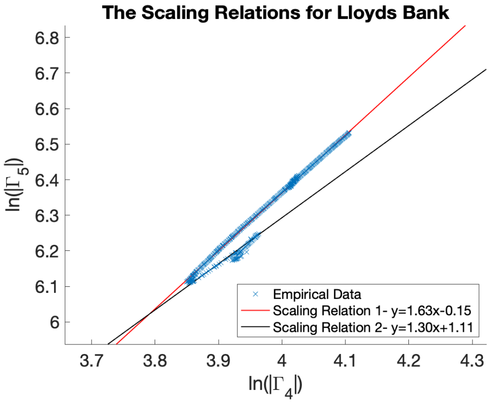

(b)

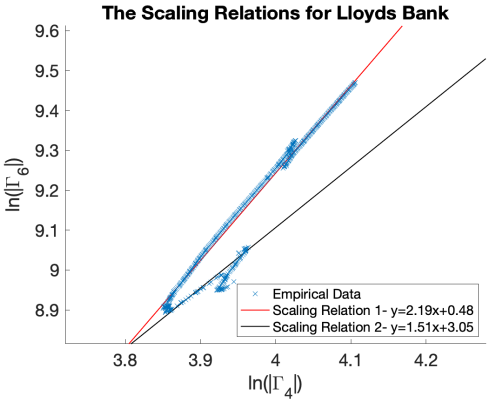

(c)

Figure B.3: We present the odd order standardised moments against even order standardised moments (panel (a) and (b)) in comparison with the fourth against the sixth order standardised moments (panel (c)), for windows greater than 2000 events for the Lloyds bank time series. In all panels, we recover the double scaling seen for the longer and shorter time windows. However, for odd order moments, we cannot present windows shorter than around 2000 events, due to the moments passing through zero and changing their sign.
